# Evaluation of the effects of pine-sourced biochar on cattle performance and methane and carbon dioxide production from growing and finishing steers

**DOI:** 10.1093/tas/txac152

**Published:** 2022-11-30

**Authors:** J L Sperber, B C Troyer, G E Erickson, A K Watson

**Affiliations:** Department of Animal Science, University of Nebraska, Lincoln, NE 68583-0908, USA; Department of Animal Science, University of Nebraska, Lincoln, NE 68583-0908, USA; Department of Animal Science, University of Nebraska, Lincoln, NE 68583-0908, USA; Department of Animal Science, University of Nebraska, Lincoln, NE 68583-0908, USA

**Keywords:** beef cattle, biochar, methane

## Abstract

A feedlot growing (77-d) and finishing (111-d) experiment was conducted to evaluate the effects of feeding biochar on steer performance, methane and carbon dioxide emissions, and carcass characteristics. Two treatments were evaluated, a control diet without biochar and the same diet with biochar included at 0.8% of dietary DM (growing) or 1.0% of dietary DM (finishing). The growing diet consisted of 40% corn silage, 40% wheat straw, 15% modified distillers grains plus solubles, and 5% supplement, with 0.8% biochar replacing fine ground corn in supplement. The finishing diet consisted of 55% high-moisture corn (HMC), 35% Sweet Bran, 5% wheat straw, and 5% supplement, with biochar replacing 1.0% HMC and added as an ingredient. Biochar was sourced from ponderosa pine wood waste (High Plains Biochar, Laramie, WY) and was 83% C with 426 m^2^/g surface area for both experiments. Crossbred steers were utilized in the growing (*n* = 160; initial BW = 363 kg; SD = 16 kg) and finishing (*n* = 128; initial; BW = 480 kg; SD = 17 kg) experiments, blocked by BW, and assigned randomly to 16 pens. Pens were assigned randomly to one of two treatments (biochar vs. control) with eight replications per treatment. Four pen replications per treatment were paired within BW block and rotated randomly through an emissions barn with two chambers (each treatment was evaluated simultaneously and for two rotations) to capture average weekly emissions of CH_4_ and CO_2_. Pen was the experimental unit and chamber was included as a fixed effect for emissions data. There were no statistical differences (*P* ≥ 0.23) in performance outcomes between treatments for the growing experiment. Dry matter intake (DMI; *P* < 0.01) and average daily gain (ADG; *P* = 0.02) were 2.2% and 5.9% lower for biochar-fed steers in the finishing experiment, respectively, resulting in a lighter hot carcass weight (*P* = 0.10) and lower calculated USDA yield grade (*P* = 0.02). Emissions of CH_4_ and CO_2_ were not affected by biochar inclusion in the growing (*P* ≥ 0.22) or finishing experiment (*P* ≥ 0.60). Results from these experiments show no indication that feeding biochar, supplemented at 0.8% (growing), and 1.0% (finishing) of the diet DM, reduces methane emissions in growing or finishing cattle.

## INTRODUCTION

Methane (CH_4_) emissions have been of growing environmental concern over the last few decades based on their contribution to climate change. Methane is emitted to the atmosphere via natural sources, such as wetlands and enteric fermentation from wildlife, and by human activities, including the petroleum and natural gas sectors and enteric fermentation from domesticated ruminant animals (NASEM, 2016). Although modern beef production resulted in a 16% decrease in carbon footprint per unit of beef compared to the 1970s (Capper, 2011), the beef industry has been further challenged to lower its contribution to greenhouse gas (GHG) emissions. The rumen serves as a fermentation vat equipped with various microbial populations allowing cattle to digest and convert plant products such as cellulose into high-quality proteins like meat and milk (Layman, 2018). Enteric CH_4_ production is critical in anaerobic fermentation and ruminal H_2_ recycling (Sharp et al., 1998), but does represent an energetic loss for the animal ranging from 2 to 12% of gross energy intake (GEI) depending on diet (Johnson and Johnson, 1995).

One proposed method to reduce CH_4_ production in cattle is by feeding a product called biochar. Biochar is produced by burning organic matter at high temperatures in the absence of oxygen (Hansen et al., 2012), resulting in a carbonized charcoal product. When included in the ruminant diet, there are several theories on the mode of action of biochar for affecting methane production, which stem from the porous nature and large surface area of the product. Biochar may adsorb CH_4_ gas in the rumen, increase the inert surface area in the rumen impacting the microbial community, or alter the rumen microbial population (Leng et al., 2013, 2014; Saleem et al., 2018). When biochar was included in vitro in high-forage diets, such as cassava root (Leng et al., 2012b) and barley silage (Saleem et al., 2018), a reduction in CH_4_ production was observed. However, when biochar was included in vitro in a combined oaten pasture, maize silage, and concentrate diet, no difference in CH_4_ production was observed (Teoh et al., 2019). Previous literature evaluating the effect of feeding biochar on enteric CH_4_ production in vivo has mixed results. Leng et al. (2012a) reported a 24% reduction in CH_4_ production from cattle fed a basal diet of cassava root chips and foliage supplemented with 0.62% biochar produced from rice husks. Winders et al. (2019) included pine-sourced biochar at 0%, 0.8%, and 3% of dietary DM in a growing and finishing experiment, reporting numerical reductions in CH_4_ production of 9.5% and 18.4% (g/kg DMI) at the 0.8% inclusion rate in the growing and finishing experiments, respectively. Biochar utilized in Winders et al. (2019) had a C content of 85%, bulk density of 88.10 kg/m^3^, and surface area of 323 m^2^/g.

Contradictory to the findings of Leng et al. (2012a), Terry et al. (2019) reported no effect of pine-sourced biochar supplemented at 0%, 0.5%, 1.0%, and 2.0% dietary DM with a basal diet of 60% barley silage and 35% barley grain on CH_4_ production (g/kg DMI). There are a broad variety of biochars available, and the characterization of the product can differ significantly (McFarlane et al., 2017) based on organic matter source, burning or processing method, management, and transport. The variability between biochars utilized throughout the literature may be a factor in the mixed results as well as differences in basal diet fed and cattle type. The objectives of the following experiments were to determine the effects of wood-sourced biochar on cattle performance, carcass characteristics, CH_4_ and CO_2_ emissions from growing, and finishing beef steers.

## MATERIALS AND METHODS

All procedures and animal management practices were approved by the University of Nebraska-Lincoln Institutional Animal Care and Use Committee (IACUC approval # 1785). Biochar is not currently approved by the U. S. Food and Drug Administration (FDA) to be fed to cattle intended for human consumption. Prior to experiment initiation, a food use authorization from the FDA was obtained for cattle utilized in this experiment to enter the human food chain.

### Growing Experiment

A 77-d feedlot growing experiment was conducted at the University of Nebraska-Lincoln (UNL) Eastern Nebraska Research, Education, and Extension Center (ENREEC) near Mead, NE, to evaluate the impact of biochar inclusion in a forage-based diet on performance and CH_4_ emissions from cattle. Biochar utilized for both the growing and finishing experiments was provided by High Plains Biochar LLC (Laramie, WY) and was sourced from ponderosa pine trees as part of forest wood waste. Biochar was processed by High Plains Biochar LLC to a small particle size to reduce opportunity of cattle sorting in the bunk. Six randomized grab samples of biochar collected through the growing and finishing periods were sent to Control Laboratories (Watsonville, CA) for chemical analysis. Dry matter of the biochar fluctuated with ambient moisture from 57% to 76% DM with an average of 70%. On a DM basis, biochar carbon content was 82.8%, with a surface area of 426 m2/g, bulk density of 107.8 kg/m^3^, total N content of 0.7% of total dry mass, and pH of 9.49. Biochar particle size distribution ranged from less than 0.5 mm to 8 mm, with approximately 66% of sampled biochar measuring less than 2 mm, and 1% of sampled biochar measuring greater than 4 mm.

Steers (*n* = 160; initial BW = 363 kg; SD = 16 kg) were assigned to two treatments (Table 1); a negative control growing diet (no biochar inclusion) and a growing diet with 0.8% biochar inclusion on a DM basis. Biochar was included at 0.8% in the growing experiment based on results from Winders et al. (2019), who demonstrated that biochar included at 0.8% of the dietary DM had the greatest numerical reduction in CH_4_ emissions when compared to 3.0% inclusion. Diets fed were identical between treatments other than the biochar inclusion, which replaced fine ground corn in the supplement. Biochar was weighed and mixed into the feed truck as an ingredient each day. Steers were stratified into 3 BW blocks, 3 reps in the light block, 4 reps in the middle block, 1 rep in the heavy block, and assigned randomly to pen (10 steers/pen). Pens were assigned randomly to treatment (*n* = 16).

**Table 1.** Composition of diet (DM) fed to steers in growing experiment (77 days on feed)

	Treatments	
Ingredient, % of diet DM	Control	Biochar
Wheat straw	40	40
Corn silage	40	40
MDGS^*a*^	15	15
Supplement^*b*^		
Finely ground corn	2.188	1.408
Biochar	–	0.800
Limestone	1.310	1.310
Tallow	0.125	0.105
Urea	1.000	1.000
Salt	0.300	0.300
Beef trace mineral	0.050	0.050
Vitamin A-D-E	0.015	0.015
Rumensin-90^*c*^	0.012	0.012
Nutrient analysis, %		
DM	65.3	65.1
OM	90.3	90.3
Crude Protein	9.7	9.7
Neutral Detergent Fiber	57.5	57.5

^*a*^MDGS = Modified distillers grains plus solubles.

^*b*^Supplement fed at 5% of dietary DM.

^*c*^Formulated to supply monensin (Rumensin-90; Elanco Animal Health; Greenfield, IN) at 200 mg/steer daily.

Prior to initiation of the growing experiment, steers were individually identified and processed upon arrival to the ENREEC research feedlot. Steers were administered a modified live vaccine for prevention of infectious bovine rhinotracheitis, bovine viral diarrhea, parainfluenza 3, bovine respiratory syncytial virus, mannheimia haemolytica, and pasteurella multocida (Vista, Merck Animal Health, Summit, NJ), a killed vaccine for clostridial toxoids and *Histophilus somni* (Ultrabac 7/Somubac, Zoetis Inc, Kalamazoo, MI), and an injectable solution for the treatment and control of gastrointestinal roundworms, lungworms, eyeworms, lice, and mites (Dectomax, Zoetis Inc.). Before experiment initiation, steers were limit-fed a common diet of 50% alfalfa hay and 50% Sweet Bran (Cargill, Blair, NE) offered at 2% of BW for 5 d to equalize gut fill (Watson et al., 2013). Steers were weighed in the morning before feeding on days 0 and 1 of the experiment and weights were averaged to establish initial BW. The same weighing protocol was used to determine ending BW, with cattle limit fed for 5 d and weighed on 2 consecutive days. Steers were implanted with 80 mg trenbolone acetate and 16 mg estradiol (Revalor-IS; Merck Animal Health) on day 1 of the experiment.

### Finishing Experiment

A 111-d feedlot finishing experiment was conducted immediately following the growing experiment, utilizing the same group of steers. Steers (*n* = 128; initial BW = 480 kg; SD = 17 kg) remained in the same treatment groups as the growing experiment, and 2 steers (lightest BW) were removed from each pen to better accommodate chamber space in the emission barn, reducing pen count from 10 to 8 steers per pen. Two treatments were evaluated in the finishing experiment (Table 2); a negative control finishing diet (no biochar inclusion) and finishing diet with 1.0% biochar inclusion, which replaced 1.0% high-moisture corn (HMC) in the ration. Diets were identical other than biochar inclusion, and contained wheat straw, HMC, and Sweet Bran (Cargill, Blair, NE). Biochar was weighed and mixed into the feed truck as an ingredient each day. Immediately following the growing experiment, steers were limit-fed a common diet of 50% alfalfa hay and 50% Sweet Bran offered at 2% of BW for 5 d to equalize gut fill. Steers were then weighed in the morning before feeding on days 0 and 1 of the finishing experiment and weights were averaged to establish initial BW for the finishing experiment and ending BW for the growing experiment. Steers were implanted with 200 mg trenbolone acetate and 20 mg estradiol (Revalor-200; Merck Animal Health) on day 1 of experiment.

**Table 2.** Composition of diet (DM) fed to steers in finishing experiment (111 days on feed)

	Treatments	
Ingredient, % of diet DM	Control	Biochar
High-moisture corn	55	54
Sweet bran^*a*^	35	35
Wheat straw	5	5
Biochar^*b*^	–	1
Supplement^*c*^		
Finely ground corn	2.879	2.879
Limestone	1.630	1.630
Tallow	0.100	0.100
Salt	0.300	0.300
Beef trace mineral	0.050	0.050
Vitamin A-D-E	0.015	0.015
Rumensin-90^*d*^	0.016	0.016
Tylan-40^*e*^	0.010	0.010
Nutrient analysis, %		
DM	69.3	69.3
OM	88.8	88.8
Crude protein	13.4	13.4
Neutral detergent fiber	24.2	24.2

^*a*^Sweet Bran = branded wet corn gluten feed produced by Cargill (Cargill corn milling, Blair NE).

^*b*^Biochar added as an ingredient to the feed truck and replaced high-moisture corn inclusion in the diet.

^*c*^Supplement fed at 5% of dietary DM.

^*d*^Monensin (Rumensin; Elanco Animal Health, Indianapolis, IN) targeted to provide 33 mg/kg dietary DM.

^*e*^Tylosin (Tylan; Elanco Animal Health) targeted to provide 90 mg/steer daily.

Feed was delivered to pens once daily at approximately 0800 h, aiming for trace amounts of feed in the bunk during time of feeding. Weekly grab samples of dietary ingredients were completed for determination of DM and as-fed proportions of ration ingredients were adjusted weekly if required. Weekly feed samples were composited by month and composites were analyzed for DM, OM, crude protein (CP), and neutral detergent fiber (NDF) content. Feed samples (analyzed in duplicate) were oven-dried at 60 °C for 48 h (AOAC International, 1999; method 4.2.03) to determine DM content. Composites from each month of the experiment were analyzed for NDF with the addition of 0.5 g of sodium sulfite (Mertens et al., 2002; Van Soest et al., 1991) and CP (LECO Corp., St. Joeseph, MI; AOAC International, 1999; method 990.03).

Cattle were adapted to the finishing diet in 4 steps over 21 d. Step 1 diets were fed from day 1 to day 5 and contained (DM-basis) 35% Sweet Bran, 31% wheat straw, 29% HMC, and 5% supplement. Step 2 diets were fed from day 6 to day 11 and contained 35% Sweet Bran, 24% wheat straw, 36% HMC, and 5% supplement. Step 3 diets were fed from day 12 to day 16 and contained 35% Sweet Bran, 17% wheat straw, 43% HMC, and 5% supplement. Step 4 diets were fed from day 17 to day 21 and contained 35% Sweet Bran, 10% wheat straw, 50% HMC, and 5% supplement. Biochar at 1.0% of the dietary DM replaced HMC in each of the above steps for the biochar treatment.

Steers were harvested at a commercial abattoir (Greater Omaha, Omaha, NE) at experiment completion. On the day of shipping, pens were offered 50% of the previous day’s feed offering at regular time of feeding. Cattle were loaded and shipped to the abattoir in the afternoon for slaughter the next morning. Hot carcass weights (HCW) were recorded on day of slaughter and USDA marbling scores, 12th rib fat thickness, and longissimus muscle (LM) area were recorded after a 48-hr chill. Calculated yield grade was determined using the following equation (USDA, 2016): 2.50 + (0.98425 × 12th rib fat, cm) + (0.2 × 2.5 KPH, %) + (0.00837 × HCW, kg) − (0.0496 × LM area, cm^2^), where KPH fat was assumed to average 2.5%. Carcass adjusted final BW was calculated from HCW divided by a common dressing percentage of 63.

### Gas Emissions

The UNL ENREEC emission barn, equipped with a negative pressure system to monitor and record CH_4_ and CO_2_ production, was utilized for 8 consecutive weeks to monitor emissions from growing steers, followed by an additional 8 consecutive weeks to monitor emissions from finishing steers. The emission barn, as described by Winders et al. (2020), has 2 isolated pens (no emission cross-over) and operates using two air sensors, the LI-COR 7500 and LI-COR 7700 (LI-COR, Lincoln, NE) to monitor CO_2_ and CH_4_, respectively. Eight pens of cattle, 4 control, and 4 biochar, were selected randomly to rotate through the methane barn by pairing replications within BW block, representing 1, 2, and 1 rep from light, middle, and heavy block, respectively. Pairings were rotated through the barn for two 5-d periods, with each treatment represented in the barn concurrently. Each week, steers entered the barn Wednesday morning and remained in the barn until Monday morning when they were returned to their respective feedlot pen. Manure CO_2_ and CH_4_ emissions were measured from the accumulation of 5 d of manure buildup and was calculated for the remainder of Monday when cattle were absent from barn. The barns were scraped clean using a skid steer each Tuesday to develop a baseline emission level post manure removal. Baseline emission levels of CO_2_ and CH_4_ were subtracted from manure emission levels of CO_2_ and CH_4_ and final values were divided over 5 d and 10 steers (growing experiment) or 8 steers (finishing experiment), to account for individual animal emissions. Following these steps, average CO_2_ and CH_4_ values of 16.89 g and 0.08 g per steer, respectively, were subtracted from the daily emissions for CO_2_ and CH_4_ in the growing experiment due to manure emissions, and average CO_2_ and CH_4_ values of 17.45 g and 0.07 g per steer, respectively, were subtracted from the daily emissions for CO_2_ and CH_4_ in the finishing experiment. Gas emissions of CH_4_ and CO_2_ were reported as g/steer and g/kg of DMI, where reported DMI (kg/d) used for the gas emission calculations were averaged from the weekly intakes of each treatment during rotation through the respective emission chambers.

### Statistical Analysis

Performance and emissions data were analyzed using the MIXED procedure of SAS (SAS Institute, Inc., Cary, NC) with pen as the experimental unit. Performance data included BW block as a fixed effect. For emissions data in the growing experiment, day was a repeated measure. During the growing experiment, 6 d (out of 40 total) were not usable for emissions measurement due to complications with the barn sensor recording. Concentrations of CO_2_ and CH_4_ reached greater than 60 ppm at certain points throughout the day, which may be beyond the capacity of the sensor for accurate measurement (Winders et al., 2020). Unexpectedly high concentrations of CO_2_ and CH_4_ in the growing experiment were due to housing 10 steers per chamber and the high inclusion of low-quality forage in the diet. Emissions data in the finishing experiment utilized chamber as a fixed effect. Due to complications with the CO_2_ analyzer, CO_2_ emissions were averaged from one replication per treatment for each period. In addition, one replication (week two of finishing experiment barn rotations) had unusable data for CH_4_ emissions. Significance was considered at α ≤ 0.05 and a tendency was considered at 0.05 < α ≤ 0.10.

## RESULTS AND DISCUSSION

### Growing Experiment

There were no statistical differences in performance outcomes, including average daily gain (ADG; *P* = 0.46), dry matter intake (DMI; *P* = 0.23), or feed efficiency (*P* = 0.25), between biochar supplemented steers and control (Table 3). Performance outcomes for the growing experiment were similar to that of previous research analyzing the impact of biochar supplementation in high-forage diets (Terry et al., 2020; Winders et al., 2019). Winders et al. (2019) reported no difference in DMI when biochar inclusion in a high forage diet (21% brome hay, 20% wheat straw, 30% corn silage, 22% wet distillers grains plus solubles) increased from 0 to 3.0%. In a backgrounding experiment by Terry et al. (2020) evaluating the inclusion of 0%, 0.5%, 1.0%, or 2.0% enhanced pine biochar in a 60% barley silage and 30% barley grain diet on steer performance, DMI and ADG were not impacted by varying inclusion levels of biochar in the diet. Additionally, Conlin et al. (2021) fed varying pine-sourced biochar inclusions at 0%, 1.0%, 2.0%, and 3.0% of diet DM to multiparous cows fed a high-forage diet (50% alfalfa haylage, 30% wheat straw, 17% corn silage) and found no impact on DMI or ADG between treatments. These results were dissimilar to Leng et al. (2012a), who reported a tendency for *Bos indicus* type cattle fed a high-forage diet supplemented with rice husk biochar to have improved live weight gain. Diet composition (specifically forage quality), type of cattle, and differences in measurement techniques may all contribute to the differing results.

**Table 3.** Effect of pine-sourced biochar addition at 0.8% of dietary DM on performance and gas emissions of growing steers

	Treatments			
	Control	Biochar	SEM	*P-*value
Performance				
Initial BW, kg	363	363	0.91	0.96
Ending BW, kg	477	479	2.04	0.50
DMI, kg/d	8.57	8.45	0.08	0.23
ADG, kg	1.45	1.47	0.023	0.46
Gain:Feed	0.170	0.175	0.003	0.25
Gas emissions				
DMI, kg/steer^*a*^	9.6	9.7	0.11	0.52
CH_4_, g/steer	196.2	203.8	6.62	0.45
CH_4_, g/kg of DMI	20.5	20.9	0.66	0.60
CO_2_, g/steer	5,725	5,982	143.1	0.25
CO_2_, g/kg of DMI	561.3	581.4	10.8	0.24

^*a*^Dry matter intake (DMI) was used to unitize reported emissions and was averaged from the weekly intakes of each treatment during rotation through the respective emission chambers.

### Finishing Experiment

Biochar-supplemented steers had a significant decrease in dry matter intake (DMI; *P* < 0.01) and average daily gain (ADG; *P* = 0.02) and tended to have a lighter carcass adjusted final BW (*P* = 0.10) compared to the control (Table 4). Feed efficiency did not statistically differ between the two treatments (*P* = 0.22). The significant reduction in DMI observed for biochar-fed steers in the finishing experiment is dissimilar to results in previous literature (Terry et al., 2020; Winders et al., 2019). Winders et al. (2019) evaluated the effects of pine-sourced biochar inclusion at 0%, 0.8%, and 3.0% of dietary DM in a finishing diet (53% dry-rolled corn, 15% corn silage, 15% wet distillers grains plus solubles) on steer performance, and observed no difference in DMI for steers supplemented with differing inclusion levels of biochar. Similarly, Terry et al. (2020) demonstrated that supplementing enhanced pine biochar at 0%, 0.5%, 1.0%, or 2.0% in a 85% barley grain and 10% barley silage finishing diet did not significantly impact DMI.

**Table 4.** Effects of pine-sourced biochar addition at 1.0% dietary DM on performance, carcass characteristics, and gas emissions of finishing steers

	Treatments			
	Control	Biochar	SEM	*P-*value
Performance				
Initial BW, kg	479	481	2.08	0.55
Carcass Adjusted Final BW^*a*^, kg	667	658	4.00	0.10
DMI, kg/d	13.4	13.1	0.06	<0.01
ADG, kg	1.69	1.59	0.03	0.02
Gain:Feed	0.126	0.122	0.002	0.22
*Carcass characteristics*				
HCW, kg	420	415	2.5	0.10
LM area, cm^2^	95.5	94.8	0.90	0.93
Marbling^*b*^	455	455	10.2	0.97
12th rib fat^*c*^, cm	1.55	1.45	0.046	0.12
Calculated yield grade	3.23	3.18	0.041	0.02
*Gas emissions*				
DMI, kg/steer^*d*^	11.8	12.0	0.55	0.59
CH_4_, g/steer	168.7	165.7	5.60	0.71
CH_4_, g/kg of DMI	15.0	14.3	0.95	0.60
CO_2_, g/steer	6,282	6,173	375	0.87
CO_2_, g/kg of DMI	589.7	523.6	143	0.80

^*a*^Carcass adjusted final BW was determined from hot carcass weight (HCW) divided by common dressing percentage of 63%.

^*b*^Marbling score: 400 = small^00^, minimum required for U.S. Low Choice.

^*c*^12th rib fat, cm: calculated by back-calculating from the USDA YG equation.

^*d*^Dry matter intake (DMI) was used to unitize reported emissions and was averaged from the weekly intakes of each treatment during rotation through the respective emission chambers.

The reduction in ADG for biochar-supplemented steers in the finishing experiment is congruent with Terry et al. (2020) who reported a tendency for enhanced pine biochar included at 2.0% of a high-grain diet to reduce overall ADG and total weight gain. This reduction in gain may be attributed to the replacement of 1.0% HMC in the current finishing experiment and 2.0% of the TMR in Terry et al. (2020), as biochar has been described as largely indigestible within the rumen (Terry et al., 2019).

Biochar-fed steers tended to be lighter in hot carcass weight (HCW; *P* = 0.10) with numerically reduced 12th rib fat (*P* = 0.12), resulting in a significantly lower USDA calculated yield grade (*P* = 0.02) compared to the control. Reduced HCW and improved yield were a function of the significant reduction in DMI and ADG for biochar-fed steers compared to the control steers. A Canadian experiment by Terry et al. (2020) reported no significant difference in HCW between steers with or without biochar supplemented in a high-grain diet; however, the numerical trend of their data showed a reduction in HCW as biochar inclusion in the diet increased, where steers fed biochar at 2.0% of dietary DM were approximately 10 kg lighter than control steers. The Canadian experiment also reported a significant reduction in USDA yield grade for cattle fed biochar at 2.0% of diet, which may be influenced by the lighter HCW of steers supplemented with biochar. Results from the finishing experiment showed no difference in LM area or marbling (*P* ≥ 0.93) which was congruent with Terry et al. (2020).

### Production of CH_4_ and CO_2_

Emissions of CH_4_ and CO_2_ did not statistically differ between steers fed biochar and control treatments for the growing experiment (*P* ≥ 0.24; Table 3) or the finishing experiment (*P* ≥ 0.60; Table 4). Based on results from this experiment, there was no indication that feeding biochar reduced CH_4_ emissions in growing steers.

In general, diets with high quantities of forage (such as growing rations), have greater enteric CH_4_ production per unit of energy intake, than diets with high quantities of concentrate (such as finishing rations; Beauchemin and McGinn, 2006; Winders et al., 2019). Results from the current experiments were consistent with this idea, with numerically greater CH_4_ losses (reported as g/steer and g/kg of DMI) in the forage based growing experiment compared to the concentrate based finishing experiment. However, the 2 experiments were not statistically compared. Beauchemin and McGinn (2006) estimated losses due to enteric CH_4_ production as around 6% of gross energy intake (GEI) for forage-fed cattle and 3.5% for concentrate-fed cattle. Leng et al. (2012a) reported a 24% reduction in CH_4_ production from cattle native to Southeast Asia when supplemented rice husk biochar at 0.62% of a high-forage (cassava root chip) diet. Winders et al. (2019) reported numerical reductions of 9.5% and 18.4% CH_4_ production (g/kg DMI) with pine-sourced biochar inclusion of 0.8% in high-forage and high-concentrate diets, respectively. Headbox experiments are usually very accurate, but can be limited in scope of reference (Dillon et al., 2021; Gunter and Cole, 2016). Although the reductions in CH_4_ production from feeding biochar reported by Winders et al. (2019) were numerical differences, the relatively small sample size and short term measurements used in the headbox experiment encouraged us to further expand on the implications of feeding biochar in a pen setting, in the current experiment. Findings from the current experiment give more evidence that the numerical differences in the headbox experiment were not statistically or biologically relevant.

The mechanism by which dietary biochar inclusion reduces enteric CH_4_ production is based on one or more of the following theories: biochar adsorbs ruminal CH_4_ gas, biochar increases the inert surface area of the rumen resulting in greater opportunity for microbial colonization, and biochar alters the rumen microbial community (Leng, 2014; Leng et al., 2012a, 2013). The mechanism by which biochar adsorbs CH_4_ in the rumen is no longer the leading theory accepted in literature, considering that previous experiments reporting reductions in enteric CH_4_ production included biochar at <1.0% dietary DM (Leng et al., 2012a), which is seemingly not an adequate quantity of biochar to adsorb the volume of gas. The porous nature and large surface area of processed biochar supports the theory by which biochar increases the opportunity for microbial colonization, potentially resulting in an alteration of the rumen microbial community. The increase in surface area provided by dietary biochar may provide a functional site for microbial biofilm formation within the rumen (Leng, 2014). Improved biofilm formation may support improved efficiency of microbial growth and proliferation and, therefore, increased feed degradation (Leng, 2014; Leng et al., 2012a).

Dissimilar to the findings of Leng et al. (2012a), Conlin et al. (2021) fed multiparous cows a high-forage diet with pine-sourced biochar inclusions at 0%, 1%, 2%, and 3% of diet, and reported no impact on CH_4_ emissions. The CH_4_ emissions findings from Conlin et al. (2021) were similar to that of the present experiment. This was likely because the biochar utilized in Conlin et al. (2021) was of similar characterization to the biochar utilized in the present experiment, with a carbon content (as % of dry mass) of 83.6%, surface area of 456 m2/g, bulk density of 78.5 kg/m^3^, and pH of 10.5. Type of diet, physical properties of the biochar, and inclusion percentage of biochar in the diet are all potential reasons for differing emission and performance results between experiments.

In conclusion, pine-sourced biochar included at 0.8% of diet DM during the growing experiment did not impact cattle performance or CH_4_ emissions; however, during the finishing experiment, biochar inclusion at 1.0% diet DM reduced DMI and ADG, resulting in a tendency for reduced HCW and a significant improvement in lean carcass yield grade, with no impact on CH_4_ emissions.
